# *AoBck1* and *AoMkk1* Are Necessary to Maintain Cell Wall Integrity, Vegetative Growth, Conidiation, Stress Resistance, and Pathogenicity in the Nematode-Trapping Fungus *Arthrobotrys oligospora*

**DOI:** 10.3389/fmicb.2021.649582

**Published:** 2021-06-22

**Authors:** Meihua Xie, Jiangliu Yang, Kexin Jiang, Na Bai, Meichen Zhu, Yingmei Zhu, Ke-Qin Zhang, Jinkui Yang

**Affiliations:** ^1^State Key Laboratory for Conservation and Utilization of Bio-Resources in Yunnan, and Key Laboratory for Southwest Microbial Diversity of the Ministry of Education, Yunnan University, Kunming, China; ^2^School of Resources, Environment and Chemistry, Chuxiong Normal University, Chuxiong, China; ^3^School of Life Science, Yunnan University, Kunming, China

**Keywords:** *Arthrobotrys oligospora*, cell wall integrity pathway, mitogen-activated protein kinases *Bck1* and *Mkk1*, cell wall damage, Woronin body, trap formation

## Abstract

The cell wall integrity (CWI) pathway is composed of three mitogen-activated protein kinases (MAPKs), Bck1, Mkk1/2, and Slt2, and is one of the main signaling pathways for fungal pathogenesis, cell wall synthesis, and integrity maintenance. In this study, we characterized orthologs of *Saccharomyces cerevisiae Bck1* and *Mkk1* in the nematode-trapping (NT) fungus *Arthrobotrys oligospora* by multiple phenotypic comparison, and the regulation of conidiation and cell wall synthesis was analyzed using real-time PCR (RT-PCR). Both Δ*AoBck1* and Δ*AoMkk1* mutants showed severe defects in vegetative growth, cell nucleus number, and stress resistance. Both the mutants were unable to produce spores, and the transcription of several genes associated with sporulation and cell wall biosynthesis was markedly downregulated during the conidiation stage. Further, cell walls of the Δ*AoBck1* and Δ*AoMkk1* mutants were severely damaged, and the Woronin body failed to respond to cellular damage. In particular, the mutants lost the ability to produce mycelial traps for nematode predation. Taken together, *AoBck1* and *AoMkk1* play a conserved role in mycelial growth and development, CWI, conidiation, multi-stress tolerance, trap formation, and pathogenicity. We highlighted the role of *AoBck1* and *AoMkk1* in regulating the Woronin body response to cellular damage and cell nucleus development in *A. oligospora*.

## Introduction

The fungal cell wall is essential to maintain cellular structure and protect the cells from environmental stresses; its integrity is very important for fungal survival and adaption under adverse condition (Levdansky et al., 2010). The cell wall integrity (CWI) pathway is an important and conserved mitogen-activated protein kinase (MAPK) signaling pathway commonly found in fungi, which senses the cell wall stress signals produced by fungal cells during normal growth or environmental changes and responds in time (Turrà et al., 2014). The CWI pathway is one of the main regulatory pathways for fungal cell wall synthesis and integrity maintenance and is also related to the pathogenicity of pathogens (Hohmann, 2002). In yeast, the CWI pathway controls cell wall synthesis and cell cycle progression through a conserved MAP kinase cascade (Bck1, Mkk1/2, and Slt2) in response to cell surface stress; its signal transmission depends on the cascade phosphorylation reaction, where protein kinase C first activates the phosphorylation of Bck1, and Bck1 activates the phosphorylation of MKK1/2 which finally triggers the phosphorylation of Slt2, thereby transmitting signals to downstream effectors (Levin, 2005, 2011; Jin et al., 2015).

In recent years, the CWI pathway has also been characterized in several filamentous fungi, such as *Aspergillus fumigatus* (Valiante et al., 2009; Levdansky et al., 2010), *Magnaporthe grisea* (Zhao et al., 2005; Jeon et al., 2008), and *Beauveria bassiana* (Luo et al., 2012; Chen et al., 2014). These studies indicated that after the cascade components of the CWI pathway were destroyed in fungi, the mutant strains showed varying degrees of cell wall defects and increased their sensitivity to cell wall interfering agents, which affected their pathogenicity. In addition, the components of the CWI-MAPK cascade are also involved in a variety of biological processes, such as mycelial growth, conidiation, and environmental stress response. In *Saccharomyces cerevisiae*, *Slt2* plays an important role in regulating the cell cycle and promoting cell polarization (Zarzov et al., 1996). Similarly, the homologous gene of *Slt2* is necessary for conidiation in several filamentous fungi such as *A. fumigatus* (Valiante et al., 2009) and *Metarhizium robertsii* (Chen et al., 2016). *Slt2* also plays a role in osmotic pressure regulation in several fungi, such as *B. bassiana* (Chen et al., 2014), *M. robertsii* (Chen et al., 2016), and *Botrytis cinerea* (Liu et al., 2011). Evidently, the CWI-MAPK signaling pathway cascade participates in the regulation of multiple biological processes in different pathogenic fungi.

Nematode-trapping (NT) fungi are an important natural enemy of nematodes. The vegetative hyphae of NT fungi can specifically form a variety of traps (such as adhesive networks, adhesive knobs, and constricting rings) to capture and infect nematodes (Su et al., 2017; Yang et al., 2020). The trapping initiates a series of processes, including adhesion, penetration, and immobilization of nematodes (Nordbring-Hertz, 2004; Yang et al., 2011). Trap formation is a prerequisite for NT fungi to capture nematodes and is also an important indicator for their lifestyle switch from a saprophytic to a predacious stage (Nordbring-Hertz et al., 2001; Su et al., 2017). *Arthrobotrys oligospora* is a species of NT fungi which usually forms adhesive networks (traps), and when prey, such as nematodes, appear in the environment, the vegetative hyphae of *A. oligospora* specialize to form adhesive networks for nematode predation (Nordbring-Hertz, 2004). In 2011, we sequenced the genome of *A. oligospora*, and proteomic analysis suggested that the G protein signaling pathway might be involved in trap formation and pathogenicity (Yang et al., 2011). Subsequently, Yang et al. (2020) proved that G protein signaling plays an indispensible role in trap formation and pathogenicity of *A. oligospora*. Genomic analysis showed that there is a conserved MAPK cascade pathway in *A. oligospora* that is homologous to Bck1-Mkk1-Slt2 in yeast. Recently, we found that orthologous *Slt2* plays an important role in two NT fungi, *A. oligospora* and *Monacrosporium haptotylum* (syn. *Dactylellina haptotyla*), which is involvement in mycelial growth and stress resistance. Specifically, the deletion of *Slt2* abolished conidiation and trap formation (Zhen et al., 2018). Similarly, a *Slt2* deletion mutant of *A. oligospora* displayed severe defects in vegetative growth, conidiation, and trap morphogenesis (Chen et al., 2021). However, the functions of *Bck1* and *Mkk1* upstream of *Slt2* are poorly understood in *A. oligospora* and NT fungi specifically. In this study, orthologous *A. oligospora Bck1* (*AoBck1*) and *Mkk1* (*AoMkk1*) were characterized *via* multi-phenotypic analyses, and their regulatory mechanisms in conidiation and stress resistance were analyzed by real-time PCR (RT-PCR).

## Materials and Methods

### Fungal Strains, Plasmid Vector, and Culture Conditions

*Arthrobotrys oligospora* (ATCC24927) and the mutant strains Δ*AoBck1* and Δ*AoMkk1* were cultured on potato dextrose agar (PDA) plates at 28°C. *Saccharomyces cerevisiae* strain FY834 (ATCC90845) was cultured in YPD (10 g/L yeast extract, 20 g/L peptone, and 20 g/L glucose) broth or solid YPDA medium (YPD with 16 g/L agar). Plasmids pRS426 and pCSN44 were used to construct the recombinations, and they were maintained in the *Escherichia coli* strain DH5α (TaKaRa, Shiga, Japan). PDAS medium (PDA supplemented with 0.6 M sucrose) was used for protoplast regeneration, and cornmeal-molasses-yeast (CMY), tryptone-glucose (TG), and tryptone yeast-extract glucose agar (TYGA) media were used to analyze mycelial growth and related phenotypic traits, as previously described (Ma et al., 2020). *Caenorhabditis elegans* (strain N2) worms were incubated in an oatmeal medium at 26°C for the bioassays (Xie et al., 2020).

### Analysis of the *AoBck1* and *AoMkk1* Sequence

The sequences of *AoBck1* (AOL_s00054g475) and *AoMkk1* (AOL_s00076g699) were retrieved based on the homologous sequences of the model fungi *S. cerevisiae*, *A. nidulans*, and *N. crassa* by performing BLASTP searches in the NCBI database. Homologous sequences of *AoBck1* and *AoMkk1* in diverse fungi were searched using the BLAST algorithm in GenBank, and the DNAman software was used to align and examine their similarity. The Expasy-Compute pI/MW tool^1^ was used to calculate the theoretical isoelectric point (*p*I) and molecular weight (MW) of *AoBck1* and *AoMkk1*.

### Disruption of *AoBck1* and *AoMkk1* Genes

The *AoBck1* and *AoMkk1* genes were disrupted using homologous recombination, as previously described (Tunlid et al., 1992; Colot et al., 2006). Briefly, 5' and 3' flanking sequences of the target gene and the hygromycin-resistance gene cassette (*hph*) were amplified from *A. oligospora* and pCSN44 with paired primers (Supplementary Table S1), respectively. Then, three DNA fragments and linearized pRS426 (digested with *EcoRI* and *XhoI*) were co-transformed into *S. cerevisiae* using electroporation. Finally, the recombinant plasmids, pRS426-AoBck1-hph and pRS426-AoMkk1-hph, were individually transformed into *A. oligospora* protoplasts, as described previously (Yang et al., 2018). Southern blots and PCR analyses were used to further verify the transformant colonies grown on the PDAS medium (Tunlid et al., 1999). The Plant Genomic DNA Kit (TaKaRa) was used to extract genomic DNA from fungal strains, and their DNA were individually digested with restriction enzymes *Sph*I and *Nhe*I for Southern blot analysis. The North2South Chemiluminescence Hybridization and Detection Kit (Pierce, Rockford, IL, United States) was used for the Southern blots according to the manufacturer’s instructions.

### Comparison of Mycelial Growth, Morphology, and Conidiation

The wild-type (WT) *A. oligospora* strain and the mutant strains Δ*AoBck1* and Δ*AoMkk1* (two independent transformants were used for phenotypic analysis) were cultured on PDA plates at 28°C for 6 days, following which 7 mm diameter hyphal discs of each strain were inoculated separately in PDA, TYGA, and TG media at 28°C for 3–7 days, and the mycelial growth rate and colony morphology were observed at specific time intervals (Xie et al., 2019). To assess the conidiation capacity of each strain, the fungal strains were cultured on CMY medium at 26°C for 15 days, and the conidial yield was determined as previously described (Liu et al., 2017). The hyphae of the WT and mutant strains were stained with 20 μg/ml calcium fluoride fluorescent white (CFW; Sigma-Aldrich, St. Louis, MO, United States) to observe the mycelial morphology. To observe the mycelial septum and cell nucleus, the hyphae of the WT and mutant strains were stained with 20 μg/ml 4′,6-diamidino-2-phenylindole (DAPI; Sigma-Aldrich, St. Louis, MO, United States) for 30 min, then washed three times with phosphate buffer (pH 6.8–7.2), and further stained with 80 μg/ml CFW for 5 min. The samples were observed using an inverted fluorescence microscope (Carl Zeiss, Heidenheim, Germany). Mycelial morphology was observed *via* scanning electron microscopy (SEM) 7 days post PDA culture (Quanta-200; FEI, Hillsboro, OR, United States), and cell wall damage and the Woronin body of hyphae were further examined *via* transmission electron microscopy (TEM; Hitachi, Tokyo, Japan) 28 h post PD culture, respectively. Samples for the SEM and TEM were treated as previously described (Zhang et al., 2013, 2019b).

### Stress Assays

Hyphal discs (7 mm diameter) of each strain were incubated on TG medium alone (control) or supplemented with chemical stressors like Congo red and SDS (cell wall interfering agents), NaCl and sorbitol (osmotic agents), H_2_O_2_ (oxidant), and menadione. The colonies were cultured at 28°C for 6 days, and the relative growth inhibition (RGI) rate of each strain was calculated on different media to evaluate the response to chemical stress, as previously described (Liu et al., 2017; Zhen et al., 2018). In addition, the fungal strains were incubated on PDA medium at 28°C for 2 days, then placed at different temperatures, such as 28, 34, 38, 40, 42, and 44°C for 8 h, and then left to grow at 28°C until the 6th day. The colony diameter was measured, and RGI values were calculated as described above. All the stress assays for each strain were repeated thrice.

### Trap Formation and Nematocidal Activity Analyses

Hyphal discs (7 mm diameter) of each strain were incubated on water agar (WA, 20%) medium at 28°C for 5 days. Then, approximately 300 wild-type L4-stage *C. elegans* nematodes were added to each WA plate to induce trap formation and nematode predation. A microscope (Olympus, Tokyo, Japan) was used to observe and count the traps and the captured nematodes from each plate at 48 h. The bioassays for each strain were repeated three times.

### Transcriptional Analyses of Selected Genes

The hyphae of the WT and mutant strains were cultured on TYGA at 28°C, and the mycelia samples were collected on the 3rd, 5th, and 7th day, and the total RNA of mycelia samples was isolated using an AxyPrep multisource RNA miniprep kit (Axygen, Jiangsu, China), and then reverse-transcribed into cDNA using a FastQuant RT kit with gDNase (TaKaRa). The cDNA samples of each strain were used as templates for RT-PCR analysis with specific paired primers (Supplementary Table S2). The β-tubulin gene of *A. oligospora* was used as an internal standard, and the transcriptional level of the genes associated with conidiation and cell wall synthesis was calculated using the 2^−ΔΔCt^ method (Livak and Schmittgen, 2001). Three replicates were performed in the RT-PCR experiments, and the RT-PCR analysis for each gene was repeated three times. The relative transcript level (RTL) of candidate genes was estimated as the transcript ratio of each mutant vs. the WT.

### Statistical Analyses

GraphPad Prism version 8.0 (GraphPad Software, San Diego, CA, United States) was used for statistical analysis of data from three repeated experiments, and the variance between the WT and mutants was estimated by the Tukey’s honest significant difference (HSD) test. Value of *p* < 0.05 was considered significant.

## Results

### Sequence Analyses of *AoBck1* and *AoMkk1*

The nucleotide sequences of *AoBck1* (8,518 bp with four introns) and *AoMkk1* (4,525 bp with four introns) were retrieved from the fungus *A. oligospora*. *AoBck1* encodes a polypeptide of 1,816 amino acids with a predicted MW of 198 kDa and a *p*I of 8.31. *AoMkk1* encodes a polypeptide of 478 amino acids with a predicted MW of 52 kDa and *p*I of 9.09. Both the encoded proteins contain multiple conserved domains and motifs, such as the protein kinase-like domain superfamily (IPR011009), protein kinase domain (IPR000719), ATP binding site of protein kinase (IPR017441), and active site of Ser/Thr protein kinase (IPR008271). It was found that both *AoBck1* and *AoMkk1* contain the conserved active site motif “-D[L/I/V] K-,” *AoBck1* contains the conservative motif “-G[S/T][V/P][F/M][W/Y]M[A/S]PE-” (Supplementary Figure S1A), and *AoMkk1* contains the conservative motif “-[S/T] xxx [S/T] in addition to “-D[L/I/V] K-” (Supplementary Figure S1B). Moreover, *AoBck1* shares a high degree of similarity (79.4 and 85.5%) with the orthologs of the NT fungi *Drechslerella stenobrocha* (Liu et al., 2014) and *D. haptotyla* (Meerupati et al., 2013), respectively. Similarly, *AoMkk1* also shares a high identity (89.4 and 93.1%) with the orthologs of NT fungi *D. stenobrocha* (Liu et al., 2014) and *D. haptotyla* (Meerupati et al., 2013), respectively. In contrast, 21.1–59.3% identity was found between *AoMkk1*/*AoBck1* and the orthologs of other filamentous fungi.

### Verification of the Positive Transformants of *AoBck1* and *AoMkk1*

Single-gene disruption mutants of *AoBck1* and *AoMkk1* were constructed by homologous recombination. The positive transformants were verified by PCR amplification with primers YZBck1-F/R and YZMkk1-F/R (Supplementary Table S1; Supplementary Figures S2A,B), respectively. To avoid the presence of false positives due to multi-site insertion or incorrect insertion sites, further identification was performed using Southern blots. Only one specific hybridization band was identified in the Δ*AoBck1* and Δ*AoMkk1* mutants, respectively, indicating that there was no non-specific recombination in the Δ*AoBck1* and Δ*AoMkk1* strains (Supplementary Figures S2Aa-c,Ba-c). Finally, two transformants for *AoBck1* and *AoMkk1* were confirmed to contain the correct mutations. A single strain from each mutant was selected for subsequent studies because the independent mutant strains for each gene showed similar phenotypic properties.

### *AoBck1* and *AoMkk1* Regulate Mycelial Growth, Morphology, and Cell Nucleus Number

**Figure 1:**
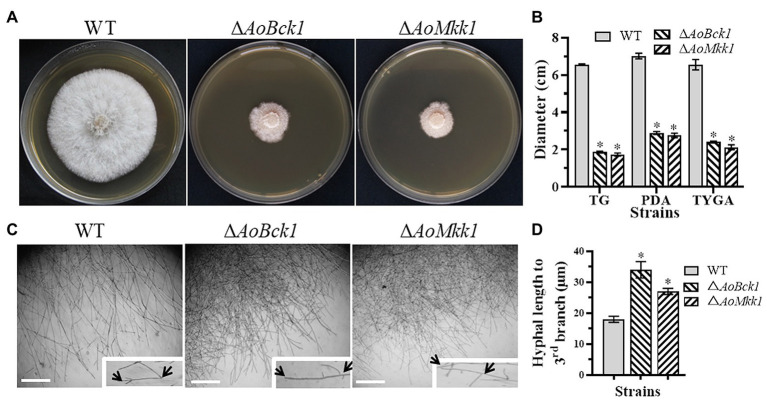
Comparison of colonial and hyphal morphologies between the wild-type (WT) and mutants. **(A)** Colony morphology of the WT and mutants incubated on tryptone yeast-extract glucose agar (TYGA) medium for 5 days at 28°C. **(B)** Colony diameters of the WT and mutants incubated on potato dextrose agar (PDA), TYGA, and tryptone-glucose (TG) media for 7 days. Error bars: SD from three replicates; asterisk: significant differences between mutant and WT (*p* < 0.05). **(C)** Mycelial morphologies of the WT and two mutants as observed by light microscopy. Arrows: mycelial tip and the third branch of mycelium. Bar = 20 μm. **(D)** The distance from the tip of the mycelium of each strain to the third branch.

**Figure 2:**
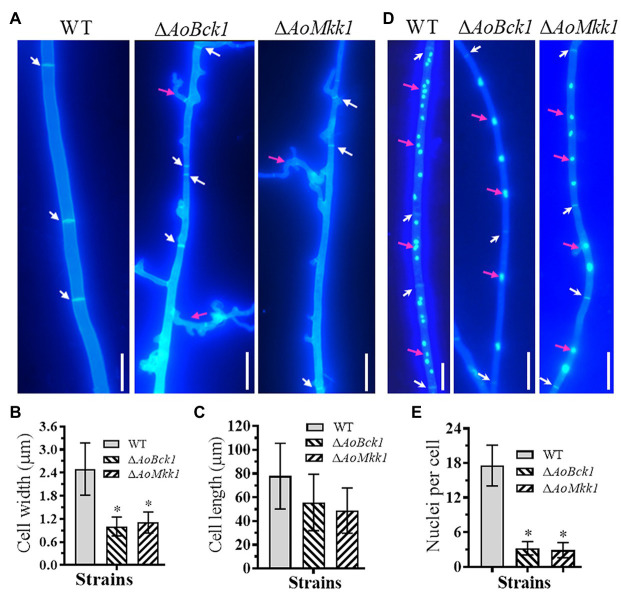
Comparison of hyphal septum, branch, and cell nucleus between the WT and mutants. **(A)** Hyphal septa of the WT and mutants were stained with 20 μg/ml calcofluor white (CFW) after the fungal strains were incubated on cornmeal-molasses-yeast (CMY) medium for 7 days. White arrows: hyphal septa; red arrows: hyphae branch. Bar = 10 μm. **(B)** Comparison of mycelial width of each strain. **(C)** Comparison of mycelial cell length of the WT and mutants; 61 mycelial cells were randomly selected, and the width and length of hyphal cell were measured using ImageJ software. Error bars: SD from 61 replicates. **(D)** Hyphae of the WT and each mutant strain were stained with CFW and 4',6-diamidino-2-phenylindole (DAPI) after the fungal strains were grown for 7 days on CMY medium; samples were examined using an inverted fluorescence microscope. White arrows: septa; red arrows: cell nucleus. Bar = 10 μm. **(E)** Comparison of mycelial cell nuclei between the WT and each mutant strain. Thirty hyphal cells were randomly selected for counting cell nuclei. Error bars: SD from 30 replicates; an asterisk indicates a significant difference compared to the WT (*p* < 0.05).

Deletion of *AoBck1* and *AoMkk1* caused a significant reduction in mycelial growth in the PDA, TG, and TYGA media (Figures 1A,B). In addition, the WT strain had very dense aerial hyphae in the TYGA medium, whereas colonies of the Δ*AoBck1* and Δ*AoMkk1* mutants were irregular and lacked aerial hyphae (Figure 1A). In addition, the mycelial tip of the third branch in the Δ*AoBck1* and Δ*AoMkk1* mutants was longer than that of the WT strain (Figures 1C,D). After CFW staining, it was found that the hyphal widths of the Δ*AoBck1* and Δ*AoMkk1* mutant strains were significantly smaller, curly short branches were increased, and the hyphal cell length was significantly uneven compared to the WT strain (Figures 2A–C). Moreover, the nuclei of the WT and the Δ*AoBck1* and Δ*AoMkk1* mutant strains were observed by DAPI staining, and the number of nuclei in the mycelial cells of the Δ*AoBck1* and Δ*AoMkk1* mutant strains was significantly reduced (only 2–4 nuclei per cell), whereas the hyphal cells of the WT strain contained 6–22 nuclei (Figures 2D,E).

### *AoBck1* and *AoMkk1* Are Necessary for Conidiation

**Figure 3:**
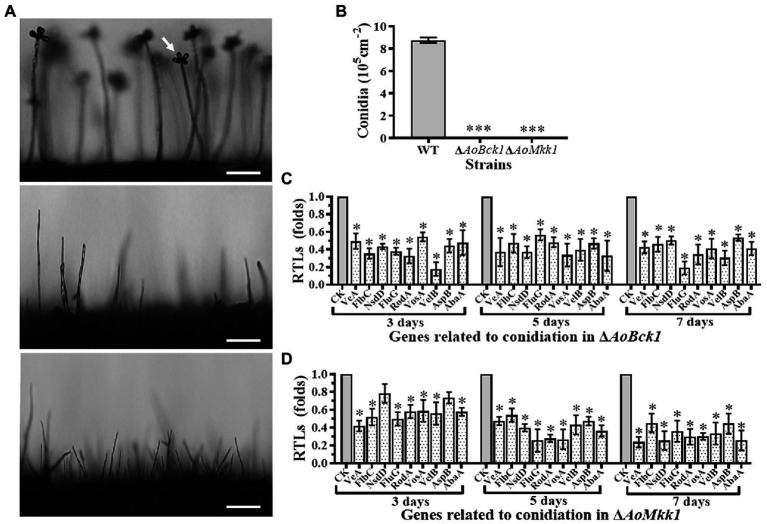
Comparison of conidiation and transcript of sporulation-related genes between the WT and mutants. **(A)** Comparison of conidiophore and sporulation the WT and mutants on CMY medium. White arrows: conidium; Bar = 10 μm. **(B)** Conidial yields in the WT vs. Δ*AoBck1* and Δ*AoMkk1* mutants. Three asterisks: very significant difference between mutant and the WT (*p* < 0.001). **(C)** Relative transcript levels (RTLs) of selected genes involved in conidiation of the WT and Δ*AoBck1* mutants at different time points. **(D)** RTLs of selected genes involved in conidiation of the WT and Δ*AoMkk1* mutants at different time points. An asterisk indicates a significant difference between the mutants and the WT strain (*p* < 0.05). CK under given conditions, using CK with an RTL of 1 as the standard, statistical analysis was performed on the RTL of each gene in each mutant and the WT strain. An asterisk indicates a significant difference compared to the WT (*p* < 0.05).

Deletion of *AoBck1* and *AoMkk1* resulted in a deleterious effect in conidiation, and two mutants were unable to produce conidia (Figures 3A,B). In order to probe into the regulation of *AoBck1* and *AoMkk1* for conidiation in *A. oligospora*, nine sporulation-related genes, *VeA*, *FlbC*, *NsdD*, *FluG*, *RodA*, *VosA*, *VelB*, *AspB*, and *AbaA*, were selected, and their transcription was analyzed at different growth stages using RT-PCR. The fungus *A. oligospora* usually begins to form conidiophores for conidiation on the 3rd day, the number of conidiophores and conidia increase rapidly on the 5th day, and most conidiophores and conidia are produced on the 7th day. The expression levels of all genes were significantly downregulated in the Δ*AoBck1* mutant (Figure 3C). Similarly, all genes involved in sporulation, except *NsdD* and *AspB* at days 3, were significantly downregulated in the Δ*AoMkk1* mutant (Figure 3D). Specifically, *VelB* and *FluG* were downregulated more significantly at day 3 and day 7, respectively, in the Δ*AoBck1* mutant (Figure 3C). Our analysis indicated that *AoBck1* and *AoMkk1* play important roles in the conidiation of *A. oligospora*.

### *AoBck1* and *AoMkk1* Play a Crucial Role in CWI

**Figure 4:**
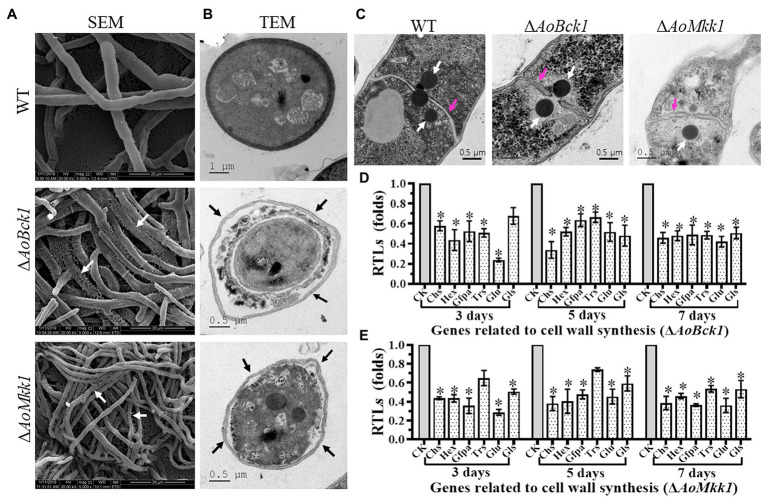
Observation of mycelial morphology using scanning electron microscopy (SEM) and transmission electron microscope (TEM), and transcript of genes associated with cell wall biosynthesis in the WT and mutants. **(A)** Mycelia of each strain were examined using SEM. Bar = 20 μm. White arrows: the fragmented mycelial surface; **(B)** Observation of hyphal cells by TEM. Bar = 1 μm (WT) or 0.5 μm (mutants). Black arrow: separation of cell wall and cell membrane. **(C)** Comparison of Woronin bodies between the WT and mutants. The hyphae of each strain cultured at 28°C in PD broth for 18 h, after which, post fixing in 2.5% glutaraldehyde, samples were examined by TEM. Red arrow: hyphal septum; white arrows: Woronin body. Bar = 0.5 μm. **(D)** RTLs of selected genes involved in cell wall synthesis of the WT and Δ*AoBck1* mutant at different time points. **(E)** RTLs of selected genes involved in cell wall synthesis of the WT and Δ*AoMkk1* mutant at different time points. CK under given conditions, using CK with an RTL of 1 as the standard, statistical analysis was performed on the RTL of each gene in each mutant and the WT strain. An asterisk indicates a significant difference compared to the WT (*p* < 0.05).

Scanning electron microscopy observations showed that the mycelial surface of the Δ*AoBck1* and Δ*AoMkk1* mutants was severely damaged, and the mycelia was fragmented and meshed (Figure 4A). Further TEM observation also revealed a similar phenomenon; the cell walls of the Δ*AoBck1* and Δ*AoMkk1* mutants were loosely distributed outside the cell membrane; and their cell membranes were incomplete. In contrast, compact cell walls and distinctly outlined membranes were observed in the WT hyphae cells (Figure 4B). To further detect cell wall damage, the WT and the mutant strains Δ*AoBck1* and Δ*AoMkk1* were cultivated in PD broth for 24 h and the mycelia of each strain were treated with cell wall-degrading enzymes, snailase, and cellulase. As a result, the hyphal cells of the Δ*AoBck1* and Δ*AoMkk1* mutants released 3.5–3.7 and 6.04–6.12-fold more protoplasts than that of the WT strain after 3 and 6 h treatments, respectively (Supplementary Figure S3). Moreover, three to four independent Woronin bodies were observed near the hyphal septa of the WT strain, and the injured hyphal septa were sealed by Woronin bodies (Figure 4C). The Woronin bodies are reduced in the mutant strains, although they are near the opening of the septa (Figure 4C). Six genes of *A. oligospora* involved in cell wall biosynthesis were transcriptionally compared between the WT and mutant strains by RT-PCR, and it was observed that all the genes were downregulated in the Δ*AoBck1* and Δ*AoMkk1* mutants compared to the WT strain (Figures 4D,E).

### *AoBck1* and *AoMkk1* Regulate Multiple Stress Responses

**Figure 5:**
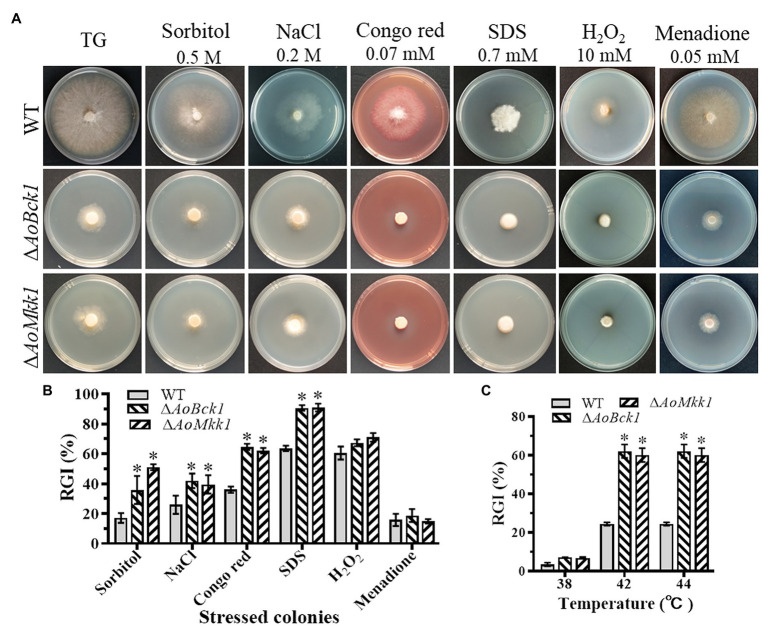
Comparison of stress response between the WT and mutants. **(A)** Colonial morphology of fungal strains under stresses of osmotic, oxidative, and cell wall interfering agents stress. **(B)** Relative growth inhibition (RGI) of fungal colonies after 7 days grown at 28°C on TG plates supplemented with 0.2 M NaCl, 0.5 M sorbitol, 0.07 mM Congo red, 0.7 mM SDS, 10 mM H_2_O_2_, and 0.05 mM menadione. **(C)** RGI values of the WT and each mutant grown at different temperatures. An asterisk indicates a significant difference compared to the WT (*p* < 0.05).

Compared to the WT, the Δ*AoBck1* and Δ*AoMkk1* mutants showed increased sensitivity to several chemical stressors like two cell wall interfering agents (Congo red and SDS) and two osmotic agents (NaCl and sorbitol), whereas they were unaffected by oxidants H_2_O_2_ and menadione (Figure 5A). The RGI values of the Δ*AoBck1* mutant exposed to 0.2 M NaCl, 0.5 M sorbitol, 0.07 mM Congo red, and 0.7 mM SDS were increased by 15.27, 30.60, 28.5, and 27.1%, respectively, as compared to the WT strain. At similar exposure concentrations of the chemical stressors as the Δ*AoBck1* mutant, the RGI values of the Δ*AoMkk1* mutant colonies with sorbitol, NaCl, Congo red, and SDS were increased by 16.02, 20.32, 33.5, and 27.4%, respectively, in comparison to the WT (Figure 5B). Moreover, the heat shock results showed that the colony growth of the Δ*AoBck1* and Δ*AoMkk1* mutants was significantly inhibited at 42°C compared to the WT (Figure 5C; Supplementary Figure S4).

### *AoBck1* and *AoMkk1* Are Required for Trap Formation and Pathogenicity

**Figure 6:**
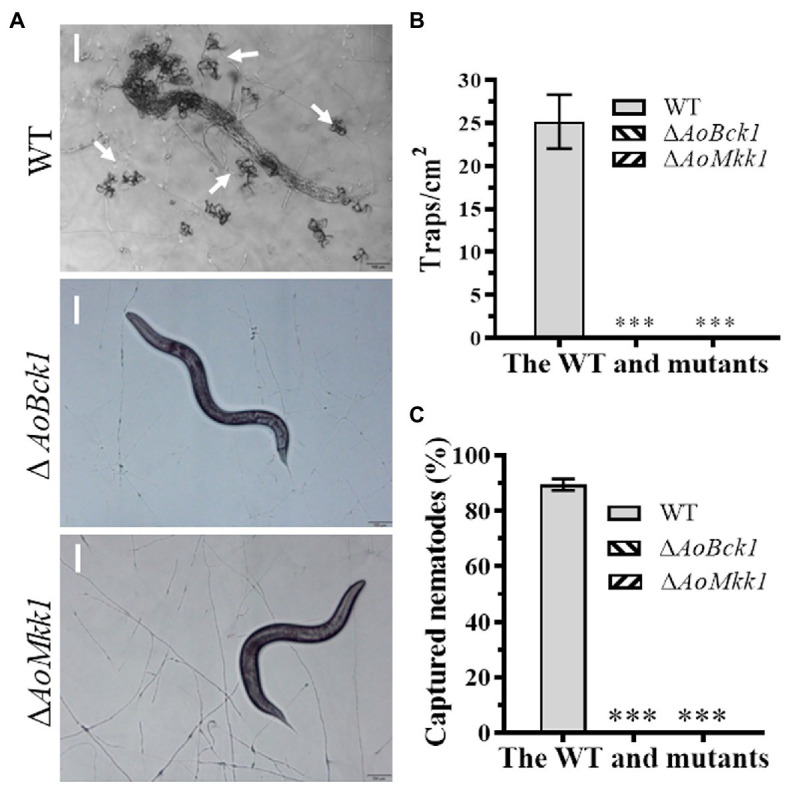
Comparison of trap formation and nematocidal activity between the WT and mutants. **(A)** Trap formation of fungal strains induced by nematodes at 48 h. White arrows: Traps. Bar: 50 μm. **(B)** Comparison of traps produced by the WT and mutants at 48 h. **(C)** Comparison of captured nematodes by the WT and mutant strains at 48 h. The asterisk indicates a significant difference compared to the WT (*p* < 0.05).

The WT strain began to produce immature traps containing 1–2 hyphal loops 12 h post nematode induction, and mature traps composed of multiple hyphal loops began to form at 24 h. Almost, all the added nematodes were captured by the WT strain at 36 h and were digested at 48 h. The WT strain produced approximately 25 traps cm^−2^ at 48 h, whereas the Δ*AoBck1* and Δ*AoMkk1* mutants did not produce any traps (Figures 6A,B). Interestingly, approximately 90.1% of the nematodes were captured by the WT strain at 48 h, whereas no nematodes were captured by the Δ*AoBck1* and Δ*AoMkk1* mutants (Figure 6C).

## Discussion

Nematodes and fungi depend on and communicate through elaborate networks of signaling. The MAPK signaling cascades are key evolutionarily conserved signal transducers in all eukaryotes, and several MAPKs have been identified in *A. oligospora*, including Slt2 (Zhen et al., 2018; Chen et al., 2021), Fus3 (Chen et al., 2021), Hog1 (Kuo et al., 2020), and Ime2 (Xie et al., 2020). In filamentous ascomycetes, the Slt2-MAPK cascade pathway is an important signaling pathway, which consists of *Bck1*, *Mkk1/2*, and *Slt2* in *S. cerevisiae* (Levin, 2005). In this study, we characterized the orthologs of *Bck1* and *Mkk1* (*AoBck1* and *AoMkk1*) in the NT fungus *A. oligospora*. It was found that deletion of *AoBck1* or *AoMkk1* affects diverse phenotypic traits in *A. oligospora*.

In several filamentous fungi, deletion of *Bck1* and *Mkk1/2* caused defects in mycelial growth and development. For example, the Δ*Bck1*, Δ*Mkk2*, and Δ*Mpka* mutants showed significantly reduced hyphal growth, elevated branching, and abnormal hyphal structure in *A. fumigatus* (Valiante et al., 2009). In *Magnaporthe oryzae*, the Δ*Mps1* mutant had severe defects in aerial hyphal growth (Xu et al., 1998). In *A. oligospora*, deletion of *AoSlt2* resulted in a serious defect in mycelial growth and aerial hyphae development in *A. oligospora* (Zhen et al., 2018; Chen et al., 2021). Similar to the Δ*AoSlt2* mutant, the Δ*AoBck1* and Δ*AoMkk1* mutants also showed a significant reduction in mycelial growth and aerial hyphae, where the colonies of the Δ*AoBck1* and Δ*AoMkk1* mutants became irregular and hyphal branches were increased compared to the WT strain. Importantly, deletion of *AoBck1* and *AoMkk1* resulted in a significant reduction in the cell nucleus number. Thus, *AoBck1* and *AoMkk1* play a conserved role in the regulation of mycelial growth and aerial hyphae in diverse fungi, and they also regulate cell nucleus number in *A. oligospora*.

*Arthrobotrys oligospora* is a typical NT fungal species that can reproduce asexually by producing abundant conidia (Zhang et al., 2019b). In this study, the Δ*AoBck1* and Δ*AoMkk1* mutants completely lost their ability for conidiation, which is similar to the Δ*AoSlt2* mutant (Zhen et al., 2018; Chen et al., 2021). Similar defects in conidiation were also found in other fungi, such as the significantly reduced conidial yields of the Slt2-MAPK cascade mutants in *M. robertsii*. Moreover, deletion of *Bck1*, *Mkk1*, and *Slt2* caused a decrease in the number of conidia in *M. oryzae* (Xu et al., 1998; Zhao et al., 2005; Jeon et al., 2008). Regarding the defect in conidiation, the transcription of genes associated with conidiation was substantially downregulated in the Δ*AoBck1* and Δ*AoMkk1* mutants compared to the WT strain. *AbaA* is a central developmental regulator in the conidiation of *A. nidulans* and other filamentous fungi (Park and Yu, 2012; Krijgsheld et al., 2013). Deletion of *AbaA* abolished aerial conidiation and submerged blastospore formation *in vitro* despite no negative impact on hyphal growth in various media in *B. bassiana* (Zhang et al., 2019a). Moreover, we found that the orthologous gene of *VelB* (*AoVelB*) is essential for conidiation in *A. oligospora*, as the Δ*AoVelB* mutant is unable to produce spores (Zhang et al., 2019b). These results indicate that *Bck1*, *Mkk1*, and *Slt2* play a conserved role in conidiation in *A. oligospora* and other fungi.

In recent years, an increasing number of studies have suggested that *Bck1*, *Mkk1/2*, and *Slt2* play a crucial role in fungal cell wall synthesis and integrity maintenance (Xu et al., 1998; Hohmann, 2002; Chen et al., 2014). In this study, deletion of *AoBck1* and *AoMkk1* caused severe damage to the cell wall of *A. oligospora*. Interestingly, protoplasts released by the Δ*AoBck1* and Δ*AoMkk1* mutants were significantly increased after treatment with cell wall-lysing enzymes. Similarly, the Δ*Bck1*, Δ*Mkk1*, and Δ*Slt2* mutants released more protoplasts after treatment with cell wall-lysing enzymes, and their cell walls become thinner or more electron-transparent in *B. bassiana* (Chen et al., 2014). In *M. oryzae*, *Mps1*, *Bck1*, and *Mkk1* were necessary to maintain CWI, and their mutants were highly sensitive to cell wall-degrading enzymes (Xu et al., 1998; Zhao et al., 2005; Jeon et al., 2008). Interestingly, the transcripts of several genes related to cell wall biosynthesis were significantly downregulated in the Δ*AoBck1* and Δ*AoMkk1* mutants, which was consistent with the phenotypic alterations. In addition, the Woronin body is a peroxisome-derived dense-core vesicle that is unique to several genera of filamentous ascomycetes (Momany et al., 2002), which is involved in sealing septal pores in response to cellular damage (Navarro-Espíndola et al., 2020). The Woronin body of *A. oligospora* also plays an important regulatory role in conidiation, trap formation, stress resistance, and adaptation to nutrient-deficient environments (Liang et al., 2017). In this study, although Woronin bodies seemed to be present in the mutants, they were unable to seal the damaged septal pores and failed to respond to cellular damage. These results indicate that the Slt2-MAPK cascade pathway plays a conserved role in regulating CWI. Furthermore, *AoBck1* and *AoMkk1* are involved in regulating the normal response of the Woronin body to cell damage.

It is important to adapt to altered environments for the vegetative growth, development, and reproduction in yeast and filamentous fungi. The Δ*AoBck1* and Δ*AoMkk1* mutants were more sensitive to some chemical stressors than the WT strain but were unaffected by the oxidants used in this study. Similar to our observation, the Δ*Bck1*, Δ*Mkk1*, and Δ*Slt2* mutants of *B. bassiana* showed high cell sensitivity to NaCl and sorbitol, but negligible responses to oxidative stress by menadione or H_2_O_2_ were observed (Chen et al., 2014). Our previous study found that the sensitivity of the Δ*AoSlt2* and Δ*MhSlt2* mutant strains to osmotic agents increased, but their sensitivity to oxidants also increased (Zhen et al., 2018). In addition, the Δ*AoBck1* and Δ*AoMKK1* mutants were more sensitive to heat shock. Similarly, deletion of *Slt2* in *B. bassiana* also resulted in increased cell sensitivity to high temperatures (Luo et al., 2012). These results show that *AoBck1* and *AoMkk1* regulate multi-stress tolerance in *A. oligospora* and other fungi.

The traps are an important feature for *A. oligospora* and other NT fungi to capture nematodes and are essential for their survival and virulence (Nordbring-Hertz, 2004). Deletion of *AoBck1* and *AoMkk1* in *A. oligospora* resulted in the complete loss of the ability to form traps and the inability to capture nematodes, which is consistent with a previous report regarding *AoSlt2* (Zhen et al., 2018; Chen et al., 2021). Similar results were also found in other fungi, such as the virulence of the Δ*Bck1*, Δ*Mkk1*, and Δ*Slt2* mutant strains being significantly reduced in *B. bassiana* (Luo et al., 2012; Chen et al., 2014) and *M. robertsii* (Chen et al., 2016). In *M. oryzae*, the Δ*Bck1* mutant was nonpathogenic to susceptible rice seedlings (Jeon et al., 2008), the Δ*Mkk1* mutant lost the ability to infect the host (Zhao et al., 2005), and the Δ*Mps1* mutant was defective in appressorium penetration (Xu et al., 1998). Moreover, deletion of one or two components of the CWI-regulated MAP kinase also impaired the virulence (Mehrabi et al., 2006; Jiang et al., 2018). These results show that CWI-regulated MAP kinases play a crucial role in the pathogenicity of diverse pathogens.

**Figure 7:**
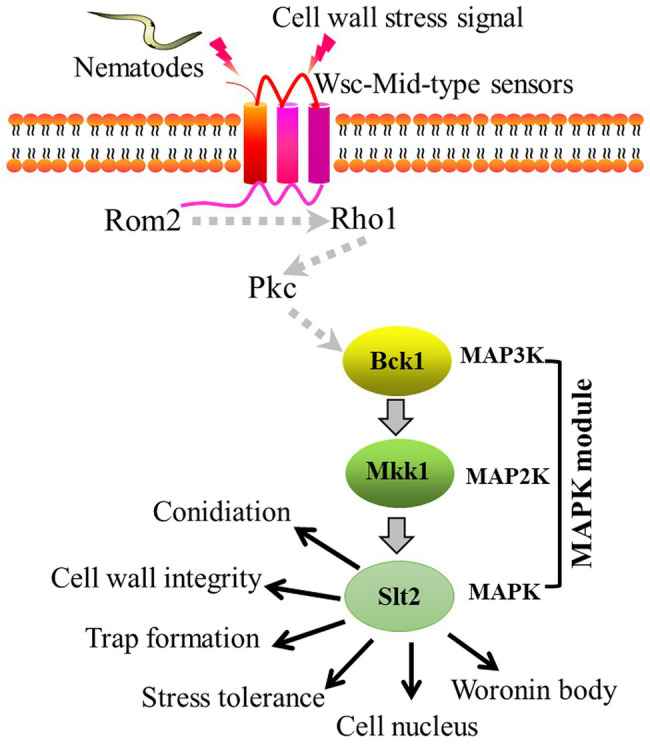
Proposed model for the regulation of *AoBck1* and *AoMkk1* in *Arthrobotrys oligospora*. *Arthrobotrys oligospora* senses nematode or cell wall stress signals and transmits them into the cells to activate Rho1 and Pkc, then triggers the cell wall integrity (CWI)-MAPK cascade and initiates various regulatory pathways, thereby regulating various a series of biological processes. The dashed arrows indicate the putative connections; the solid arrow indicates the experimental results.

In this study, we found that *AoBck1* and *AoMkk1* not only participate in the regulation of several important functions, such as CWI, conidiation, multi-stress tolerance, and pathogenicity, but also regulate additional biological processes in *A. oligospora*, including the Woronin body response to cellular damage and cell nucleus development. Accordingly, we illustrated a schematic model for the regulation of *AoBck1* and *AoMkk1* in *A. oligospora* (Figure 7). *Arthrobotrys oligospora* perceives nematode or cell wall stress signals through receptors at the surfaces of the cell membrane (Rsm), which are transferred into the intracellular environment and interact with the guanine nucleotide exchange factor Rom2 to activate the small GTPase Rho1, followed by activation of the protein kinase Pkc (Su et al., 2017; Heinisch and Rodicio, 2018), which triggers the CWI-MAPK cascade and drives the transcription of downstream genes associated with multiple phenotypic traits. However, the role of Rsm, Rho1, and Pkc should be further verified. Our results lay a good foundation for revealing the mechanisms of trap formation and lifestyle switching in *A. oligospora* and other NT fungi.

## Conclusion

We characterized orthologs of *S. cerevisiae Bck1* and *Mkk1* in the NT fungus *A. oligospora*. Both *AoBck1* and *AoMkk1* play an important role in vegetative growth, CWI, and stress resistance. In particular, *AoBck1* and *AoMkk1* are required for conidiation and trap formation, and they also play roles in regulation of cell nucleus development and Woronin body response to cell damage in *A. oligospora*. Our findings provide a basis for investigating the role and mechanism of MAPK in vegetative growth, CWI, cell nucleus development, and pathogenicity of NT fungi.

## Data Availability Statement

The original contributions presented in the study are included in the article/Supplementary Material, further inquiries can be directed to the corresponding author.

## Biosecurity Statement

The strains and other materials used in this study are not applicable for biosecurity.

## Author Contributions

JinY conceived and designed the study. MX and JinY wrote the manuscript. MX conducted the experiments. JiaY, KJ, NB, MZ, and YZ analyzed the data. JinY, K-QZ, and MX revised the manuscript. All authors contributed to the article and approved the submitted version.

### Conflict of Interest

The authors declare that the research was conducted in the absence of any commercial or financial relationships that could be construed as a potential conflict of interest.

## Footnotes

^1^https://www.expasy.org/resources/compute-pI-mw
