# Supplementary material for: AoBck1 and AoMkk1 Are Necessary to Maintain Cell Wall Integrity, Vegetative Growth, Conidiation, Stress Resistance, and Pathogenicity in the Nematode-Trapping Fungus Arthrobotrys oligospora
Source: Front Microbiol. 2021 Jun 22;12:649582. doi: 10.3389/fmicb.2021.649582 (PMC8258383; doi:10.3389/fmicb.2021.649582)
Supplement: Supplementary file 1 [file Data_Sheet_1.DOCX]

**Supplementary materials**

**1. Supplementary Figures**

**Figure S1.** Multiple alignment of orthologous Bck1 and Mkk1 from different fungi. A. Alignment of Bck1 from different fungi. The active site -D[L/I/V]K- and conservative motif -G[S/T][V/P][F/M][W/Y]M[A/S]PEV- are marked using underlines. B. Alignment of Mkk1 from different fungi. The active site -D[L/I/V]K- and conservative motif “-[S/T] xxx [S/T] are marked using underlines. Areas shaded in black are conserved regions (100% similarity); areas shaded in red have high degrees of homology (more than 75% similarity).


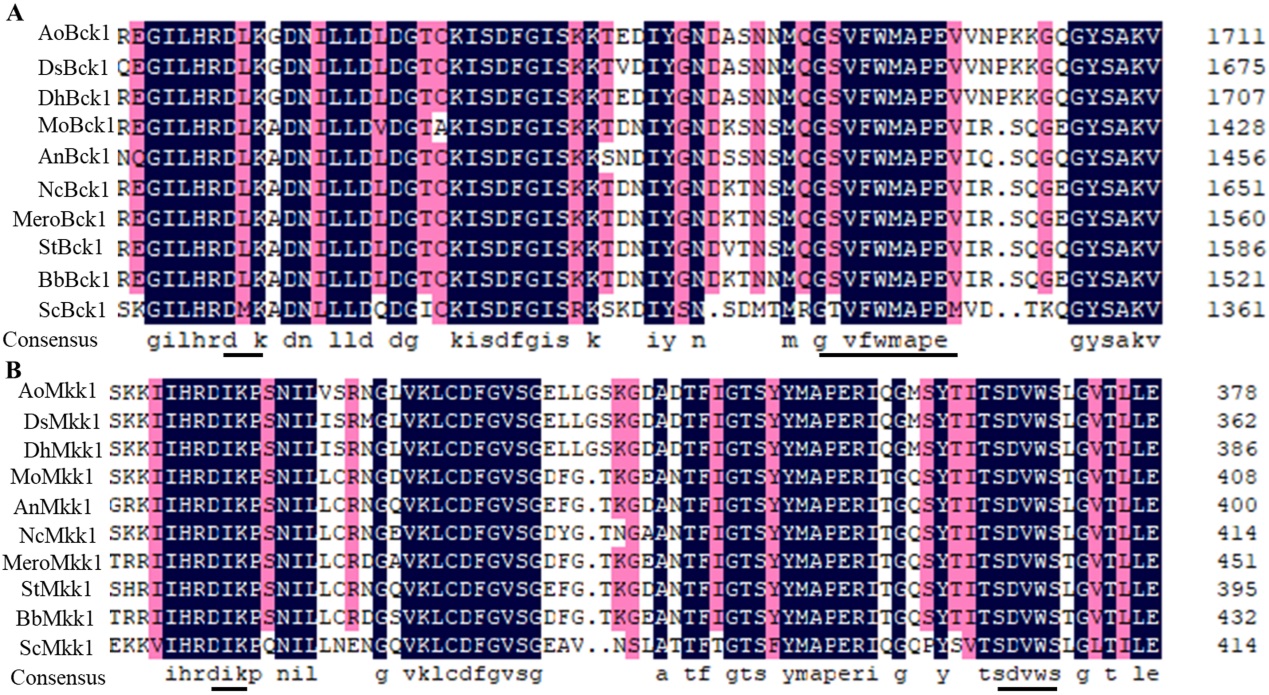


**Figure S2.** Knock-out and verification of genes *AoBck1* and *AoMkk1* in *A. oligospora*. (A) Diagrammatic sketch of homologous recombination of *AoBck1* and *AoMkk1*. The homologous flanking sequences of the target gene, Southern blot probe, and the *Kpn*I restriction enzyme sites are marked. (B) Positive transformants verified by PCR amplification. (C) The Δ*AoBck1* and Δ*AoMkk1* mutants confirmed by Southern blots.


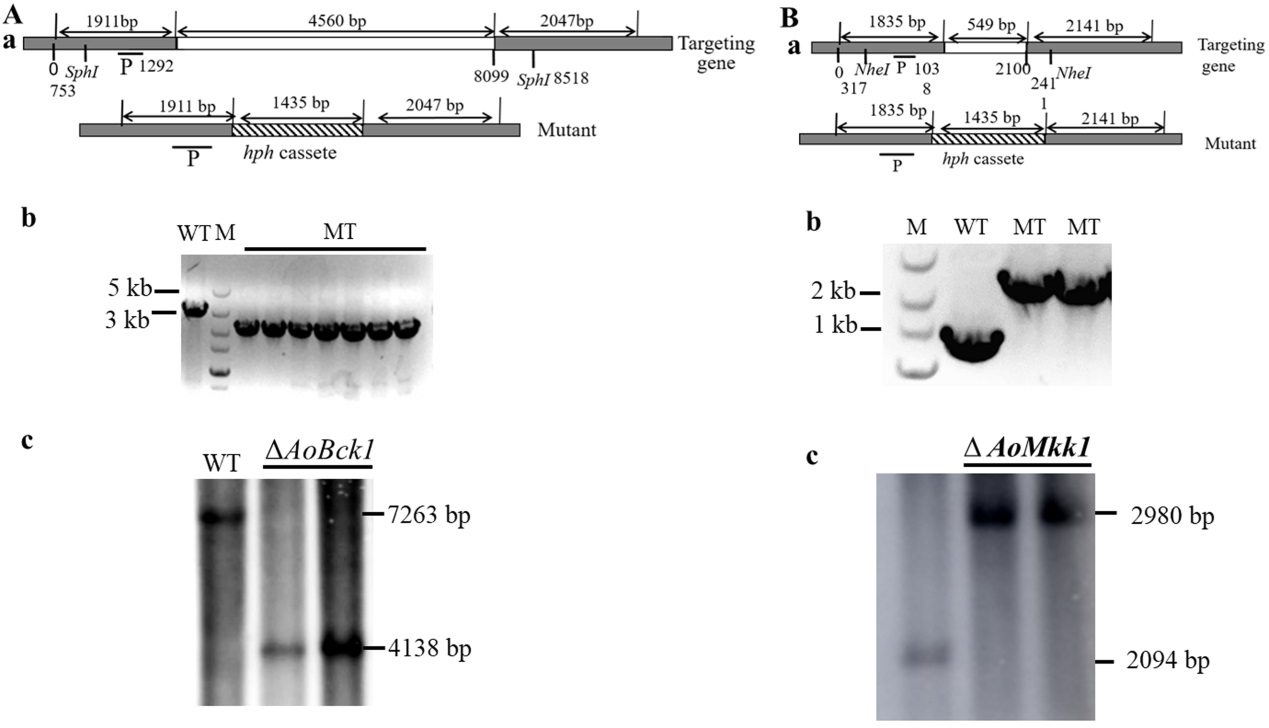


**Figure S3.** The number of protoplasts produced by the mycelia of each strain after treatment with cell wall-lysing snailase (10 mg/mL) and cellulase (12.5 mg/mL) for 3 h and 6 h.


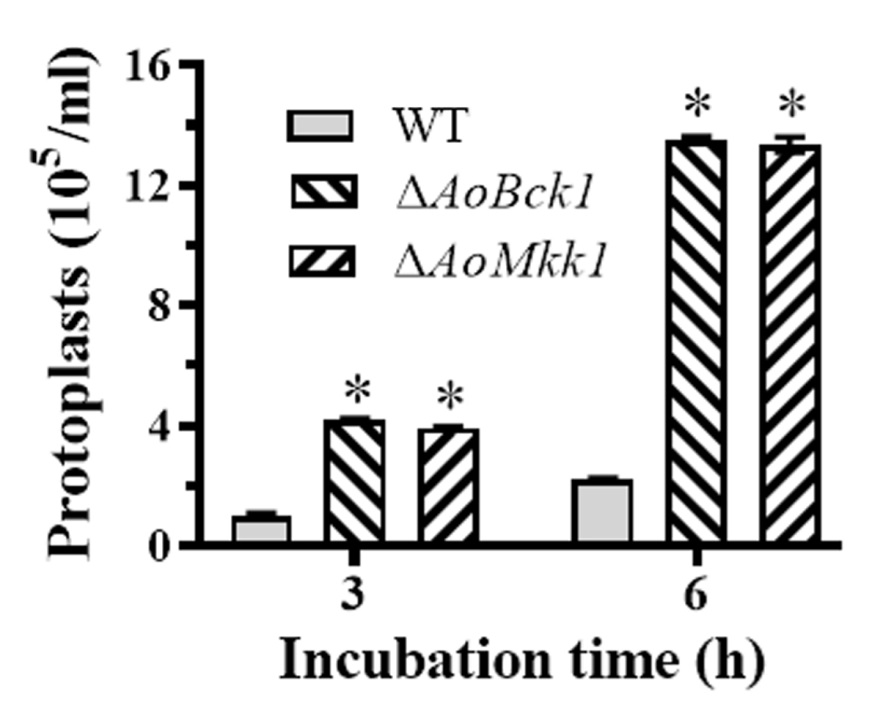


**Figure S4.** Colonial morphology between the wild-type (WT) and mutant strains under high temperature stress.


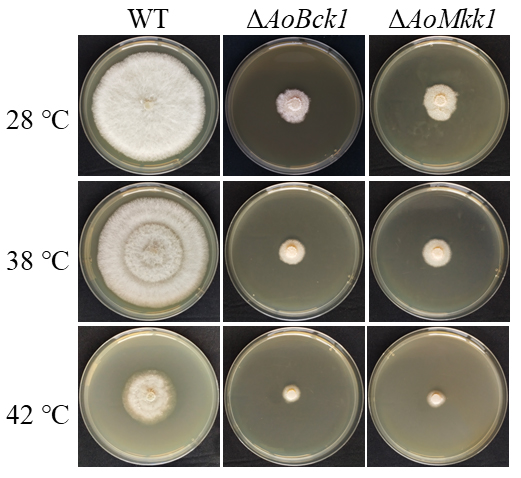


**2. Supplementary tables**

**Table S1. List of primers used in this study.**

| **primers** | **Paired sequences** | **Purpose** |
| --- | --- | --- |
| Bck1-5F/5R | CCCCTTGGTTCAATGTTCAC/GTGGATCGGTTGGGAGTTG | Amplify the 5’ fragment |
| Bck1-3F/3R | TTGCCACTTCCGTCTACGT/GCCATCCCATCATTCCTCAA | Amplify the 3’ fragment |
| YZBck1-F/R | GGCGGCATAGAAAAGACCC/GCATCAAGTCCGATAGTAGGC | Verify the transformants |
| Bck1P-F/R | GATGATGGTATCTCGGTATTT/TTCGCTTAATTGCCTGTAT | Make Southern blotting probe |
| Mkk1-5F/5R | TTCCCTATCACTACCTCACGC/TGCGACTTCGGAGTTTCTG | Amplify the 5’ fragment |
| Mkk1-3F/3R | CTCTTGAATCCTCCCATCTCG/TCAGCGGACAGGTGACATTA | Amplify the 3’ fragment |
| YZMkk1-F/R | CGTCGCTCCACTTCACTCCT/CCTCCCCAGTTCCCCAAAT | Verify the transformants |
| Mkk1P -F/R | GTCCCGTAATCAACAAGCG/AGGAGTGAAGTGGAGCGAC | Make Southern blotting probe |
| Hph-F/R | GTCGGAGACAGAAGATGATATTGAAGGAGC/GTTGGAGATTTCAGTAACGTTAAGTGGAT | Amplify the hph cassette |

**Table S2.** Paired primers used for RT-PCR analysis of genes associated with conidiation and cell wall biosynthesis in *A. oligospora*.

| **Sporulation genes** | **Paired sequences (5'-3')** |
| --- | --- |
| AOL_s00169g18(*VeA*) | AAGCTACACCCAATCAACGC/ TTGCGATGCTGACGATCTTG |
| AOL_s00007g157 (*FlbC*) | CTCTCCGGCAAAGACAATCG/ GTCGACTGAGGATAGTAGCT |
| AOL_s00075g211 (*NsdD*) | ATTACGGCCGCCTAGTAGTC/ CTCGTTTGGACCTGGTTGTG |
| AOL_s00043g361 (*FluG*) | GATTCCAGTCCCGTGAATTC/ GCTAAGGAGAGGATGGGCAT |
| AOL_s00006g570 (RodA) | GCGGATCCAACATGAAGCTT/ GGTTGACAACTGGGATGCTG |
| AOL_s00054g700 (*VosA*) | CAAACCACCCACCACCAAAT/ GGATGGACAGGAGAAGGACC |
| AOL_s00080g63 (*AbaA*) | AACTTTATGCGCCTTGTCGT/ TTGGCTAGGTGGTCTGTACG |
| AOL_s00215g893 (AspB) | ATACCGCCAACACCCTCTAC/ AACCATCTTCATCTCGGCCT |
| **Cell wall biosynthesis** | **Paired sequences** |
| AOL_s00078g76 (*Chs-3*) | GCCACTCTGCCATCTTTAGC/GCATCTTCACCCGCACCAGT |
| AOL_s00112g89 (*Hex*) | TACCCTTCTTGACCTCGCTG/ GAGACGGCGGATGAGTTTTC |
| AOL_s00076g99 (*Gfpa*) | CCATCATCGAGCACACCAAG/ CCACCGAGAGTCACTGTCTT |
| AOL_s00097g268 (*Trs*) | CACGTCCATATCACCCTCGA/ GTGGTATCGGCGACAGTTTC |
| AOL_s00083g375 (*Glu*) | GTCATCCTCAAGAACGTCGC/ ATATGGAAAGTTGGCCGTGC |
| AOL_s00054g491 (*Gls*) | AGCTCTGTTCTGGTGATGCT/ GATGTTTCGCCAAGGACTCC |
| **β-tubulin gene** | Sequence (5′-3′) |
| AOL_s00076g640 | CCACCTTCGTCGGTAACTC/ TCGTCCATACCCTCACCAG |
